# Nuclear Magnetic Resonance Approaches in the Study of 2-Oxo Acid Dehydrogenase Multienzyme Complexes—A Literature Review

**DOI:** 10.3390/molecules181011873

**Published:** 2013-09-26

**Authors:** Sowmini Kumaran, Mulchand S. Patel, Frank Jordan

**Affiliations:** 1 Department of Chemistry, Rutgers University, Newark, NJ 07102, USA; 2 Department of Biochemistry, School of Medicine and Biomedical Sciences, State University of New York at Buffalo, Buffalo, NY 14214, USA

**Keywords:** 2-oxoacid dehydrogenase complex, pyruvate dehydrogenase complex, thiamin diphosphate, protein-protein interaction, substrate channeling, lipoyl domain, peripheral subunit binding domain, NMR

## Abstract

The 2-oxoacid dehydrogenase complexes (ODHc) consist of multiple copies of three enzyme components: E1, a 2-oxoacid decarboxylase; E2, dihydrolipoyl acyl-transferase; and E3, dihydrolipoyl dehydrogenase, that together catalyze the oxidative decarboxylation of 2-oxoacids, in the presence of thiamin diphosphate (ThDP), coenzyme A (CoA), Mg^2+^ and NAD^+^, to generate CO_2_, NADH and the corresponding acyl-CoA. The structural scaffold of the complex is provided by E2, with E1 and E3 bound around the periphery. The three principal members of the family are pyruvate dehydrogenase (PDHc), 2-oxoglutarate dehydrogenase (OGDHc) and branched-chain 2-oxo acid dehydrogenase (BCKDHc). In this review, we report application of NMR-based approaches to both mechanistic and structural issues concerning these complexes. These studies revealed the nature and reactivity of transient intermediates on the enzymatic pathway and provided site-specific information on the architecture and binding specificity of the domain interfaces using solubilized truncated domain constructs of the multi-domain E2 component in its interactions with the E1 and E3 components. Where studied, NMR has also provided information about mobile loops and the possible relationship of mobility and catalysis.

## 1. Introduction

The 2-oxoacid dehydrogenase complexes (ODHc) represent the classic examples of multienzyme complexes. They have molecular masses of 4–10 MDa and they act as ‘macromolecular machines’ in which the subunit associations serve not only to co-localize the enzymes, but also couple their activities to channel substrates and products. Each ODHc occupies a key position in intermediary metabolism and their activity is under stringent control by hormones and dietary factors.

**Scheme 1:**
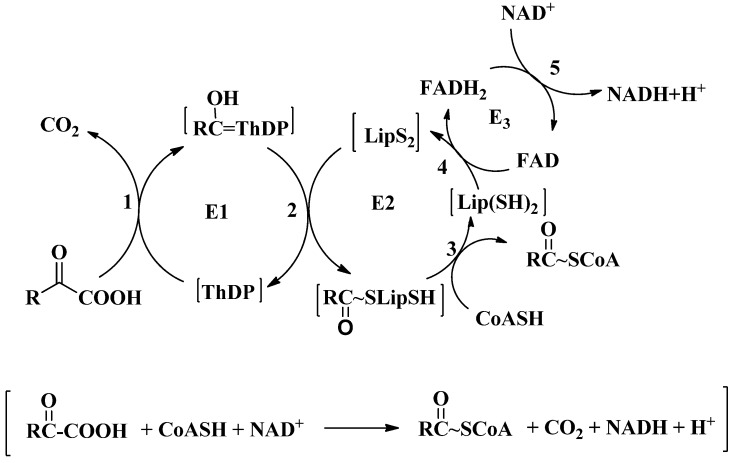
Reaction scheme of oxidative decarboxylation of 2-oxo acids by respective 2-oxo acid dehydrogenase complexes.

These complexes have a very similar design and they all catalyze the decarboxylation of 2-oxoacids to produce acyl-coenzyme A (acyl-CoA), NADH, and CO_2_ by similar coupled multistep reaction mechanisms (Scheme 1). The overall reaction is the sum of individual reactions catalyzed by the three enzyme components, and it epitomizes a classic case of substrate channeling which results in complex biotransformation. The reaction begins with the thiamin diphosphate (ThDP)-dependent irreversible decarboxylation of the 2-oxoacid catalyzed by the E1 component. The weakly acidic C2H of the ThDP thiazolium ring is converted to its conjugate base, forming a highly nucleophilic C2-carbanion/ylide/carbene, which attacks the substrate carbonyl carbon, forming a covalent tetrahedral intermediate (called a pre-decarboxylation intermediate), whose positive charge in a position β to the carboxylate moiety is an effective electrophilic center triggering decarboxylation. Loss of CO_2_ leads to the C2α-carbanion/enamine, the central intermediate in all ThDP-dependent reactions. Oxidation of the enamine by the dithiolane ring of lipoamide covalently attached to the E2 component ensues, resulting in the reductive acylation of E2 and regeneration of active E1. The E2 active site then catalyzes the transfer of the acyl moiety from dihydrolipoamide to CoA thereby producing acyl-CoA. The E3 component (an FAD/NAD^+^ dehydrogenase, shared among all ODHc’s in any cell type) re-oxidizes the dihydrolipoyl group, with concomitant reduction of NAD^+^ to NADH [1,2,3,4].

Using a ‘divide and conquer’ approach, a wealth of information has been revealed on the macromolecular structure, assembly and symmetry, active-site coupling, conformational mobility, substrate specificity and metabolic regulation of these complexes. In this review we discuss the various NMR approaches applied on these important multienzyme complexes, which provided an integrated view of many facets, from studies of organic catalysis within the protein structure to general principles governing these giant multiprotein catalytic machines.

## 2. Overview of 2-oxoacid Dehydrogenase Complexes

The large size, complexity and dynamic nature of ODHc’s have hindered X-ray structural studies of any of the intact complexes. However, the structures of the E1 and E3 components from a number of sources have been solved by X-ray crystallography [5,6,7,8,9,10,11]. The El components are homodimers in some bacterial cells, while of mammalian PDHc and BCKDHc they are α_2_β_2_ heterotetramers. The E3 component is a homodimeric FAD/NAD^+^-dependent enzyme, which is responsible for reoxidizing the dihydrolipoyl group of the lipoyl domain, the final step of the mechanism. Each E3 copy comprises four well-delineated domains: an FAD-binding domain and an NAD^+^ binding domain (both Rossmann folds), a central domain and an interface domain.

Owing to the inability so far to crystallize any intact E2 component, several three-dimensional structures have been determined for the individual domains of E2 chains [12,13,14,15,16,17,18]. The E2 component of the ODHc’s of both bacteria and eukaryotes serves as both the catalytic center for acyl-CoA production and the structural core of these multienzyme complexes and is comprised of three types of domains. Starting with the N-terminus, there are 1–3 tandem repeated lipoyl domains (LD), followed by a peripheral subunit-binding domain (PSBD) responsible for binding E1/E3 chains. The C-terminal catalytic domain (CD) provides the acyltransferase activity and oligomerizes to form an octahedral (24-mer in *E. coli* and other Gram negative bacteria) or icosahedral (60-mer in mammals and yeast and some Gram positive bacteria) inner core of the 2-oxoacid dehydrogenase complex [1]. The individual domains are separated by long, flexible linker regions allowing large movements of the lipoyl domain(s) to enable active site coupling. The number of lipoyl domains depends on the species.

The E2 components of OGDHc, BCKDHc and PDHc from Gram-negative bacteria have 24 copies arranged in octahedral symmetry [19] while PDHcs from mammals, birds, yeast and Gram-positive bacteria have 60 copies of E2 which form icosahedral symmetry [20]. In the mammalian PDHc, there are three additional components: (1) Protein X now called the E3-binding protein (E3BP) which tethers the E3 component to the E2 core [21,22]. The E3BP is homologous to E2 subunits and includes a single lipoyl domain followed by a peripheral-subunit-binding domain (PSBD) and the catalytic domain, which is devoid of transacetylase activity required to generate acetyl-CoA [23,24] (2) The activities of both mammalian PDHc and BCKDHc are subject to regulation by specific kinases (PDK, BDK) and phosphatases (PDP, BDP) [25,26,27] which control the activity of the complex by reversible phosphorylation/dephosphorylation of serine side chains in E1. A brief overview of the ODHc components is represented in Table 1.

**Table 1:** Composition of 2-oxo acid dehydrogenase complexes [28].

Enzyme	Cofactors, substrate	Species	E1	E2	E3	Kinases	Phosphatases
PDHc	ThDP, Mg^2+^, Lipoic acid, FAD, NAD^+^, CoA Pyruvate	*E. coli*	12 homodimers	24 subunits, octahedral	6 E3 dimers		-
			EC1.2.4.1	EC 2.3.1.12	EC 1.8.1.4	-	
		Human	30 E1α_2_β_2_ tetramer EC1.2.4.1	48 E2subunits +12 subunits of E3BP icosahedral EC 2.3.1.12	6 E3 dimers EC 1.8.1.4	PDK1	PDP1, PDP2
						PDK2	
						PDK3	
						PDK4	
OGDHc	ThDP, Mg^2+^ Lipoic acid, FAD, NAD^+^, CoA Oxoglutarate	*E. coli*	12 homodimers	24 subunits, octahedral	6 E3 dimers	-	-
			EC1.2.4.2	EC 2.3.1.61	EC 1.8.1.4		
		Human	12 homodimers EC1.2.4.2	24 subunits	6 E3 dimers EC 1.8.1.4	-	-
				octahedral			
				EC2.3.1.61			
BCKDHc	ThDP, Mg^2+^, Lipoic acid, FAD, NAD^+^, CoA Branched chain ketoacids	*E. coli*	12E1α_2_β_2_ tetramer	24 subunits octahedral	6 E3 dimers	-	-
			EC1.2.4.4	EC2.3.1.168	EC 1.8.1.4		
		Human	12E1α_2_β_2_ tetramer	24 subunits octahedral	6 E3 dimers	BDK	BDP
			EC1.2.4.4	EC2.3.1.168	EC1.8.1.4		

## 3. Active Site Chemistry of ThDP-Dependent Enzymes

**Scheme 2:**
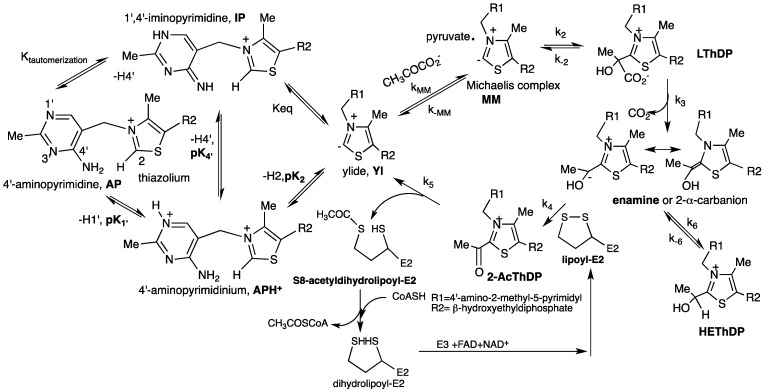
Mechanism of *E. coli* pyruvate dehydrogenase complex with role of ThDP on E1p.

The ThDP-dependent 2-oxoacid decarboxylases proceed by coenzyme-mediated electrophilic catalysis, and there are a series of covalent complexes formed between ThDP and the substrate/intermediates/product on such pathways [29,30]. ThDP assists in the catalysis of carbon–carbon bond forming and bond breaking reactions adjacent to a carbonyl group and it is composed of thiazolium and 4′- aminopyrimidine rings. ThDP as a coenzyme is unique in utilizing bifunctionality in a coenzyme: acid-base catalysis via the 4′-aminopyrimidine ring, and *umpolung* catalysis by the thiazolium ring, which together catalyze both 2-oxoacid decarboxylation and C-C carboligation reactions [31,32,33,34,35]. The ThDP can assume four ionization/tautomerization states during the enzymatic reaction: the dipositively charged N1′-protonated 4′-aminopyrimidinium (APH^+^), and the three singly positively charged forms, the 4′-aminopyrimidine (AP), the 1′, 4′-iminopyrimidine (IP) and the ylide (Yl) (Scheme 2).

The first step is ionization of a weak acid at the thiazolium C2 position to form the ThDP ylide. This ylide carries out a nucleophilic attack on the keto group of 2-oxoacids, followed by decarboxylation, forming the enamine/C2α-carbanion intermediate. This central intermediate is the branch point of catalysis and is likely present in all ThDP-dependent catalytic pathways. This enamine/C2α-carbanion can then have both non-oxidative and oxidative fates, depending on the enzyme and the presence of neighboring redox cofactors. The reaction pathway for PDHc is depicted in Scheme 2. A similar reaction mechanism is probably followed on other ODHc complexes.

Ever since the appearance of crystal structure of ThDP enzymes, the role and likelihood of tautomerization of 4′-aminopyrimidine group of ThDP to the 1′,4′-iminoThDP has gained wider acceptance [30]*.* This is suggested by two highly conserved structural features on all ThDP enzymes: (a) The V coenzyme conformation which ensures that the C2 thiazolium atom and the N4′ atom of the 4′-aminopyrimidine ring are within less than 3.5 Å from each other. It was also suggested that for such short distances, it could be difficult to fit a proton at C2 and two protons at N4′ simultaneously, and further suggesting that the 4′-aminopyrimidine ring participates in proton transfer during the reaction sequence; (b) The presence of a glutamate within hydrogen bonding distance of the N1′ atom of the 4′-aminopyrimidine ring, as a potential catalyst for the tautomerization (Scheme 2). The tautomerization reaction requires three forms of the AP ring of which two are neutral, the AP and 1′,4′-iminopyrimidine (IP), but these forms must interconvert via the positively charged, *N*1′ protonated 4′-aminopyrimidinium ion (APH^+^). The main questions concerning the molecular basis for activation of the C2-H bond of ThDP and how the enzymes can favorably stabilize the IP form during various steps of the catalytic cycle still remain unanswered [36,37,38,39,40,41,42,43]. Several NMR methods have been used to resolve such questions due to the sensitivity of NMR chemical shifts to both the ionization and tautomeric states of ThDP [44,45,46,47].

### 3.1. Characterization of the Ionization/Tautomerization States of ThDP in Models by Solution and Solid State NMR

Model studies were carried out in parallel with studies on the enzyme to understand the chemical rationale for ThDP catalysis [48]. The fortuitous acid stability of several of the ThDP-bound covalent intermediates enables both their synthesis and chemical trapping (acid quench) for eventual detection. 

**Scheme 3:**
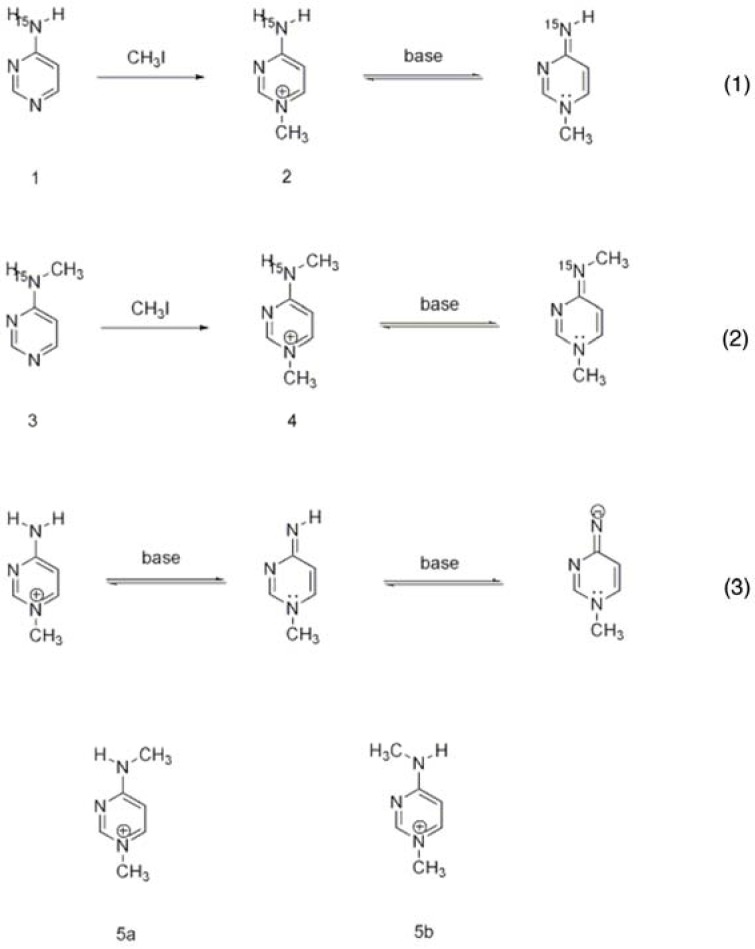
Model compounds synthesized for spectroscopic studies [45]: 4-aminopyrimidine(**1**), N1-methyl-4-aminopyrimidinium iodide (**2**), N4-methyl-aminopyrimidine (**3**), N1,N4-dimethyl-4-aminopyrimidinium iodide (**4**), N1,N4-dimethyl-4-aminopyrimidinium triflate (**5**).

Some of these compounds (Scheme 3) could then be used for generation of the 1′,4′-iminopyrimidine tautomer of the 4′-aminopyrimidine ring of ThDP. Direct ^15^N observation was achieved by DEPT [49]. Two-dimensional, one-bond proton-nitrogen correlation spectroscopy, HSQC [50,51,52], and multiple-bond proton-nitrogen correlation spectroscopy, HMBC [53,54] were recorded. These measurements were carried out in solvents of different dielectric constant, as ThDP enzymes appear to have an active center, which behaves as if it had a low apparent dielectric constant (Table 2). The ^1^H chemical shifts became more shielded on amino deprotonation of the N1-alkylpyrimidinium salts. The ^15^N experiments revealed a very large deshielding effect in the 1,4-imino tautomer compared to the amino tautomer, perhaps as large as 130 ppm. The chemical shift values enabled subsequent search for resonances in the appropriate chemical shift range on enzymes [45].

Table 2^1^H (Top) and ^15^N (Bottom) chemical shifts (*45*) of 4-aminopyrimidine (**1**), N1-methyl-4-aminopyrimidinium iodide (**2**), N4-methyl-aminopyrimidine (**3**), N1,N4-dimethyl-4- aminopyrimidinium iodide (**4**), N1,N4-dimethyl-4-aminopyrimidinium triflate (**5**).

**Table:** 

	Chemical Shifts (ppm, *vs.* TMS at 0.00 ppm)					
Compounds/Conditions	C2-H	C5-H	C6-H	N4-CH_3_	N1-CH_3_	N4-H
**1** (DMSO-d_6_)	8.31(t)	6.39(d)	8.01(d)	2.86(s)		6.79(d)
**1** (CD_3_CN)	8.47(t)	6.97(d)	7.96(d)	2.84(d)		5.63(d)
**2** (DMSO-d_6_)	8.71(t)	6.73(d)	8.17(d)	2.98(s)	3.78	8.87(dd)
**2** (CD_3_CN)	8.41(t)	6.51(d)	8.09(d)	3.02(d)	3.80	7.54(dd)
**2** + NaHMDS (CD_3_CN)	7.45(s)	5.82(d)	6.79(p)	2.81(s)	3.46	N/A
**3** (DMSO-d_6_)	8.59(s)	6.62(d)	8.04(s)	2.98		7.79(qd)
**3** (CD_3_CN)	8.41(s)	6.40(d)	8.05(s)	2.93		5.74(qd)
**4** (DMSO-d_6_)	8.81(s)	6.80(d)	8.11(dd)	2.98(s)	3.79(s)	9.42(bs)
**4** (CD_3_CN)	8.50(s)	6.99(d)	7.80(dd)	3.02(d)	3.77(s)	8.36(qd)
**4** + NaHMDS (DMSO-d_6_)	7.69(s)	5.65(d)	6.84(d)	2.81(s)	3.25(s)	N/A
**5a** (DMSO-d_6_)	8.81	6.79	8.10	2.98	3.79	9.38
**5b** (DMSO-d_6_)	8.68	6.92	8.35	2.93	3.79	8.31

**Table:** 

	Chemical Shifts (ppm, *vs*. neat NH_3_ at 0.00 ppm)	
Compounds/Method/Conditions	N4-CH_3_	N4-H
**1** (HSQC/CD_3_CN)		78.01
**2** (HSQC/CD_3_CN)		103.42
**1** (HSQC/ DMSO-d_6_)		86.13
**2** (HSQC/DMSO-d_6_)		110.60
**2** + NaHMDS (HSQC/CD_3_CN)		N/A
**3** (HSQC/CD_3_CN)		78.62
**4** (HSQC/CD_3_CN)		107.86
**3** (HSQC/ DMSO-d_6_)		78.26
**4** (HMBC/DMSO-d_6_)	107.28	
**4** + NaHMDS (HMBC/DMSO-d_6_)	212.09	
**4** + NaHMDS (HMBC/CD_3_CN)	218.02	

**Scheme 4:**
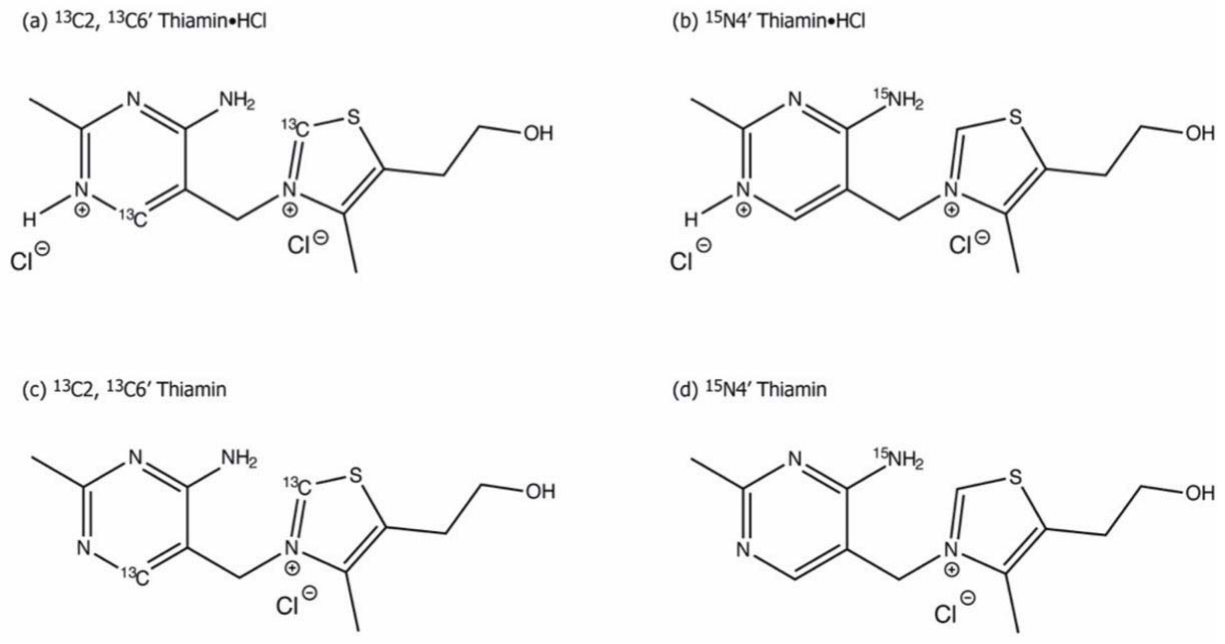
Chemical structures of Th•HCl and Th with ^13^C/^15^N enrichment at positions C2 C6′ and N4′ [46].

Later, solid-state NMR studies were carried out on model systems for different ionization and tautomerization states of thiamin (Th) and thiamin hydrochloride [46]. To achieve this, three labeled analogues [C2,C6′-^13^C_2_]Th/ Th•HCl, [C2-^13^C]Th/ Th•HCl, and [N4′-^15^N] Th/ Th•HCl) were synthesized in the Rutgers’ group (Scheme 4). Thiamin (Th) and thiamin hydrochloride (Th•HCl) correspond to the AP and APH^+^ form of ThDP, respectively.

**Figure 1:**
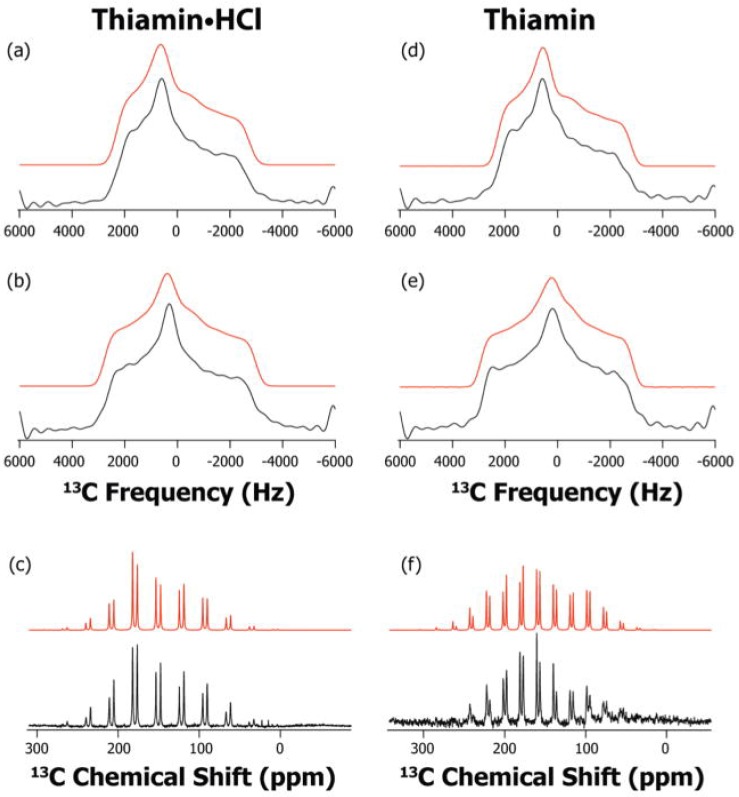
^13^C ROCSA and slow-MAS spectra of Th•HCl and Th: experimental (black) and simulated (red). (**a**) ROCSA spectrum of C2 of Th•HCl; (**b**) ROCSA spectrum C6′ of Th•HCl; (**c**) slow-MAS spectrum C2 and C6′ of Th•HCl at 2900 Hz rotor frequency; (**d**) ROCSA spectrum of C2 of Th; (**e**) ROCSA spectrum of C6′ of Th; and (**f**) slow-MAS spectrum of C2 and C6′ of Th at 2075 Hz rotor frequency [46]*.*

Magic angle spinning (MAS) NMR spectroscopy and the symmetry based recoupling of chemical shift anisotropy (ROCSA) were employed to record the ^13^C and ^15^N chemical shift anisotropy (CSA) tensors for the C2, C6′ and N4′ atoms in the model compounds (Figure.1). A DFT theoretical method was used to calculate the corresponding magnetic shielding tensors. Experimental results and DFT calculations are summarized in the solid state NMR studies of model compounds [46]. From these comparisons, it is clear that MAS NMR spectroscopy in conjunction with DFT calculations is a sensitive probe of ionization states in the thiamin cofactor. The results of this study later served as a guide for characterization of ionization and tautomeric states of ThDP when enzyme bound.

### 3.2. Characterization of ThDP-Bound Intermediates during Various Stages of the Catalytic Cycle

The large size of ThDP enzymes and the occurrence of multiple steps within the reaction sequence render it difficult to observe the reaction intermediates by solution NMR. An ingenious and powerful approach to identify and quantify the relative amount of various ThDP-bound covalent intermediates according to their C6′−H ^1^H-NMR chemical shifts after acid quench of enzyme reaction mixtures was invented by Tittmann and Hübner (TH method) [47]. This approach works efficiently as ThDP-bound covalent intermediates are typically: (i) stable under acid conditions, (ii) released into the supernatant, and (iii) can be simultaneously detected by their well-resolved C6′−H ^1^H-NMR resonances which serve as a fingerprint region for these ThDP adducts. The experiment is carried out in a chemical quench instrument. This method opens up the possibility of systematic NMR based analysis of the covalent intermediates in ThDP dependent enzymes and their active site variants.

**Figure 2:**
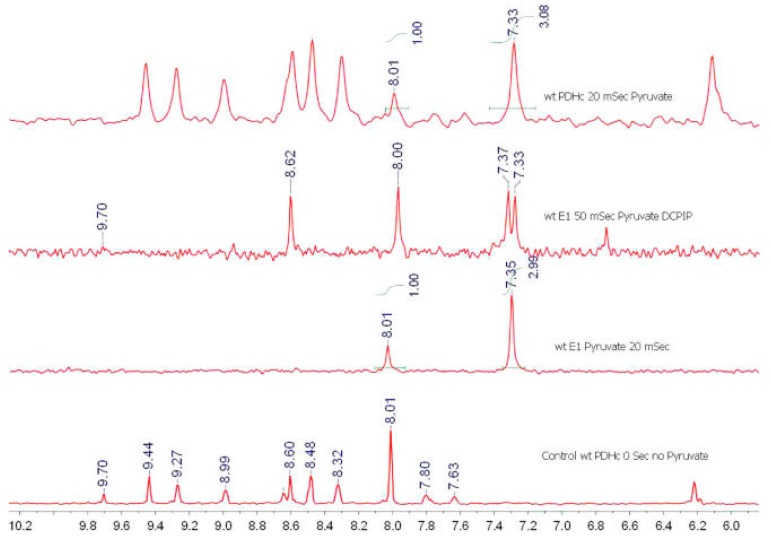
Distribution of ThDP-bound covalent intermediates in reactions of the *E. coli* E1p component and in the intact PDHc complex [55]. gCHSQC NMR spectra of the supernatant after acid quench of PDHc and removal of protein from the reaction. Top spectrum; 20 ms quench of the reaction of PDHc; Second spectrum from top: 50 ms quench of the reaction of E1p with pyruvate and DCPIP; Third spectrum from top: 20 ms quench of the reaction of E1 with pyruvate; Bottom spectrum: control, PDHc reaction mixture quenched before addition of pyruvate. ThDP-derived peaks are marked at 9.71 ppm (C2−H), 8.62 ppm (AcThDP), 8.01 ppm (C6′−H), 7.37 ppm (AcThDP), and 7.34 ppm (HEThDP). Other peaks are due to NAD^+^, CoA, DCPIP, NADH, acetyl-CoA, or DCPIPH_2_.

Based on the earlier studies on the E1 component of the *E. coli* PDHc, it was hypothesized that the dynamic regions of the E1 component undergo a disorder-order transition upon substrate binding to ThDP. This E1 loop dynamics plays a critical role in modulation of the catalytic cycle of PDHc. This mechanism could be a general feature of 2-oxoacid dehydrogenase complexes because such interfacial dynamic regions are highly conserved. In order to test this hypothesis, ThDP bound covalent intermediates on E1 and the lipoamide-bound intermediate on the E2p component were characterized and their rates of transformation were determined by using NMR and mass spectroscopy [55]*.* In an extension of the TH method, [C2,C6′-^13^C_2_]ThDP was synthesized, and using 1D ^1^H-^13^C gradient carbon heteronuclear single quantum coherence (gCHSQC) NMR experiments, protons directly attached to the two key ^13^C atoms in the aromatic region of the spectrum could be detected. An application of the method to both the E1 component and the entire PDHc complex for the detection of ThDP-related resonances even in the presence of large concentrations of aromatic substrates, products, and cofactors such as NAD^+^, NADH, CoA, acetyl-CoA, FAD, and FADH_2_ is shown in Figure 2. The method enabled assessment of the effects of complex assembly on microscopic rate constants and rate limiting steps for the first time. In experiments monitoring acetyl-CoA formation, [C3-^13^C] pyruvate was used, and the product [C2-^13^C] acetyl-CoA was again detected using the gCHSQC NMR method.

**Table 3:** Comparison of Pre-Steady–State rate constants for E1p and variants under various conditions [55].

Variant	*k_cat_* ^a^, s^−1^	*k_HEThDP_* (s^−1^)			*k_r_* (s^−1^) reductive acetylation ^c^
		Pyruvate only	Pyruvate + DCPIP	PDHc reaction ^b^	
E1p	95 ± 12	117 ± 14	73.2 ± 8.8	116 ± 25	51.7 ± 5.4
D549A	0.7 ± 0.1	12.9 ± 1.0	-	12.1 ± 0.5	-
H407A	0.08 ± 0.01	1.16 ± 0.05	0.06 ± 0.01	2.5 ± 0.19	0.02 ± 0.001
E401K	0.83 ± 0.05	0.13 ± 0.02	0.016 ± 0.003	0.59 ± 0.05	-
Y177A	3.31 ± 0.3	25.9 ± 1.2	-	-	-

^a^ The *k*_cat_ values were determined from the overall PDHc activity assay on reconstitution with E2-E3 subcomplex. ^b^ E1p-specific reaction with DCPIP assay on reconstitution with E2-E3 subcomplex. ^c^ For reaction of reductive acetylation of LD-E2p with E1p or variant and pyruvate using ESI-FT-MS.

These results suggest that formation of the first covalent pre-decarboxylation intermediate, C2α-lactylthiamin diphosphate (LThDP) is rate limiting for the series of steps, which culminate in acetyl-CoA formation. It was also revealed that substitutions in the active center loops produced variants with up to 900-fold slower rates of formation of the LThDP, demonstrating that these perturbations directly affected covalent catalysis (Table 3). This rate was recovered by up to 5-fold upon assembly to PDHc of the E401K variant. Thus for the first time, the role of loop dynamics in this covalent catalysis and the effects of complex assembly on some individual rate constants could be assessed.

### 3.3. Characterization of Ionization/Tautomerization States of ThDP on Enzymes. First Application of Solid-State NMR Spectroscopy to ThDP Enzymes

Two of the four ionization and tautomeric states of ThDP (Scheme 2, left) are well characterized via circular dichroism (CD) on several ThDP superfamily members [37,38,39]*.* However, the method is incapable of providing specific proton locations in the tautomers, which in principle may be accessible via NMR studies. The large molecular mass of ThDP enzymes (120,000–250,000 Da) has so far limited their solution NMR studies. Solid-state NMR (SSNMR) spectroscopy presents a promising alternative, which provides information that can be derived from various NMR observables with unprecedented atomic-level detail, and where there is independence of resonance line widths on the molecular size of the huge proteins [56,57,58]. To determine the precise ionization/tautomerization states of ThDP during various stages of the catalytic cycle and to probe mechanistic issues of the ThDP superfamily of enzymes, the first application of solid-state NMR spectroscopy to ThDP enzymes was carried out by the Rutgers group in collaboration with Dr. Polenova’s group at the University of Delaware [44].

**Figure 3:**
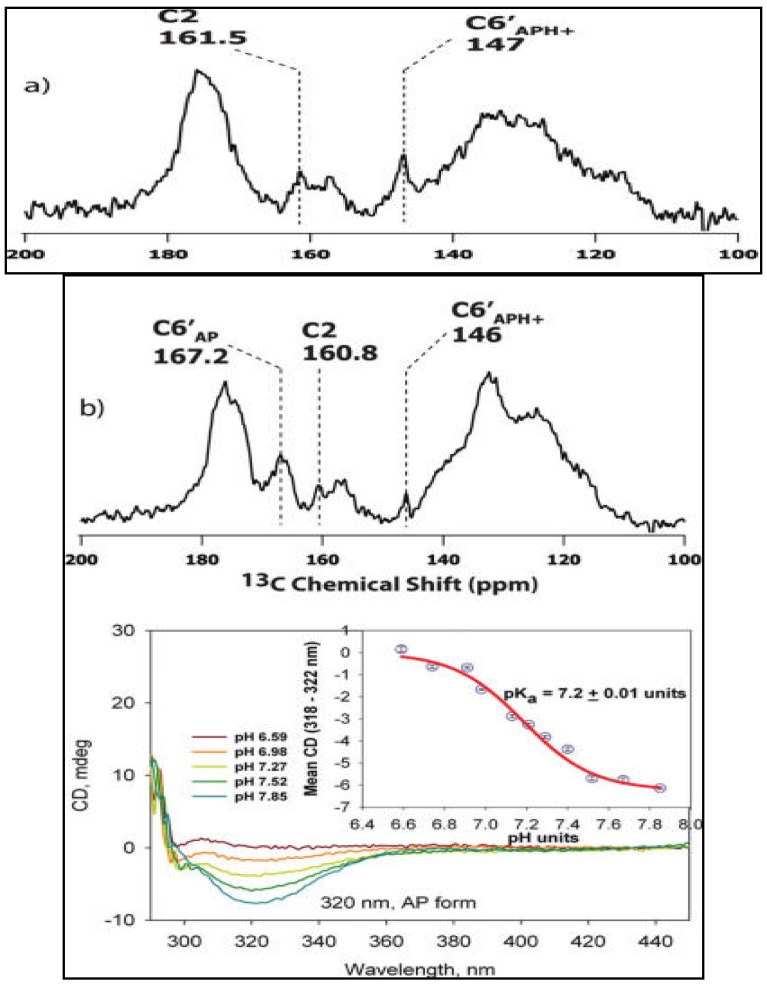
^13^C CPMAS SSNMR spectra of E1o reconstituted with [C2,C6′-^13^C_2_]ThDP [44]*.* (**a**) Spectrum acquired at pH 7.0 at 8 °C with 32,768 transients. (**b**) Spectrum acquired at pH 8.0 at 10 °C with 28,900 transients. Recycle delay was 5 s for acquiring both spectra. (Bottom). Representative near-UV CD spectra of E1o at varying pH values. E1o (5 mg/mL) in 2.4 mL of a triple buffer (15 mM MES, 15 mM KH_2_PO_4_, and 15 mM Tris) containing additional 0.5 mM ThDP and 2.5 mM Mg^2+^.

From model studies using solid-state NMR, ^13^C and ^15^N isotropic chemical shifts and complete chemical shift anisotropy tensors of key atoms (C2, C6′, and N4′) of thiamin (Table 2) in different ionization states of the 4′-aminopyrimidine ring [46] were available. With these assignments in hand, NMR results on the specifically *de novo* synthesized ThDP analogues [C2,C6′-^13^C_2_]ThDP, [C2-^13^C]ThDP, and [N4′-^15^N]ThDP could be interpreted when enzyme bound [44]. The three enzymes used in these initial studies were the E1 components of the *E. coli* pyruvate (E1p) and 2-oxoglutarate dehydrogenase (E1o) complexes and yeast pyruvate decarboxylase (YPDC). The solid-state isotropic chemical shift of the C6′ atom clearly shows that enzyme-bound ThDP is activated by protonation of the N1’ atom of the 4′-aminopyrimidine ring, elevating the pK_a_ of the APH^+^. This may be due to its electrostatic interaction with the highly conserved glutamate residue and the active site favoring a positive charge at this position. The deshielded chemical shift of the C2 atom of ThDP when bound to enzymes as compared to the free thiamin suggests increased acidic environment of the catalytic center, as well as a chemical exchange process among the different species of ThDP (Figure 3). The C2 chemical shift changes upon addition of substrate analogue provide strong evidence for the formation of tetrahedral intermediate at C2α on the enzyme. These results suggest that the enzymes activate both 4′-aminopyrimidine and thiazolium rings of the ThDP. The chemical shift of bound [N4′-^15^N]ThDP provides plausible models for the participation of the 1′,4′-iminopyrimidine tautomer in the mechanism. (For all the chemical shift values refer to [44].

3.4. ^19^F-NMR Studies on Conformational Dynamics of the E1p Loop Region

The loop regions are often thought to be conformationally less structured fragments of the protein chain, which connect two secondary structure elements. Loops exposed on the surface often play a vital role in protein functions, primarily because they have a greater chance of interacting with the solvent and other molecules. Arjunan *et al.* [59] made the important observation that in the stable pre-decarboxylation complex analogue of E1p, two active center loops (the inner one spanning residues 401–413 and the outer one spanning residues 541–557), which are not seen in the structure with ThDP alone, become clearly visible. To examine the consequences of these loops on catalysis, a cysteine-free E1p construct (E1p_-cys_; all six cysteine residues replaced per monomer) was created using site directed mutagenesis. Next, a new cysteine was engineered onto the inner loop creating the K411C E1p variant for site selective labeling for ESR, fluorescence and NMR studies.

**Figure 4:**
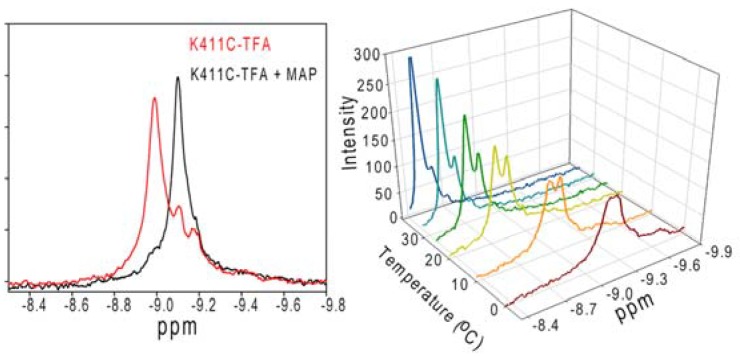
^19^F-NMR analysis of conformational dynamics of inner loop in response to MAP and temperature [60]. (**Left**) Effect of MAP addition on the spectra of K411C-TFA. The red line is a spectrum of K411C-TFA and shows three resonances at −8.993, −9.106, and −9.193 ppm. The resonance at −9.193 ppm is due to oxidized free label, did not show any ligand-induced changes, and could be removed by extended washing. The black trace is a K411C-TFA (250 µM) after addition of saturating amount (500 µM) of MAP. Spectra were acquired at 30 °C and referenced to the external standard trifluoroacetate. The data represent 2,048 transients processed with 5 Hz line broadening and a spectral window of 34,617 Hz. (**Right**) Effect of temperature on the ^19^F-NMR spectra of un-liganded K411C-TFA (250 µM). Sample conditions were the same as above.

**Figure 5:**
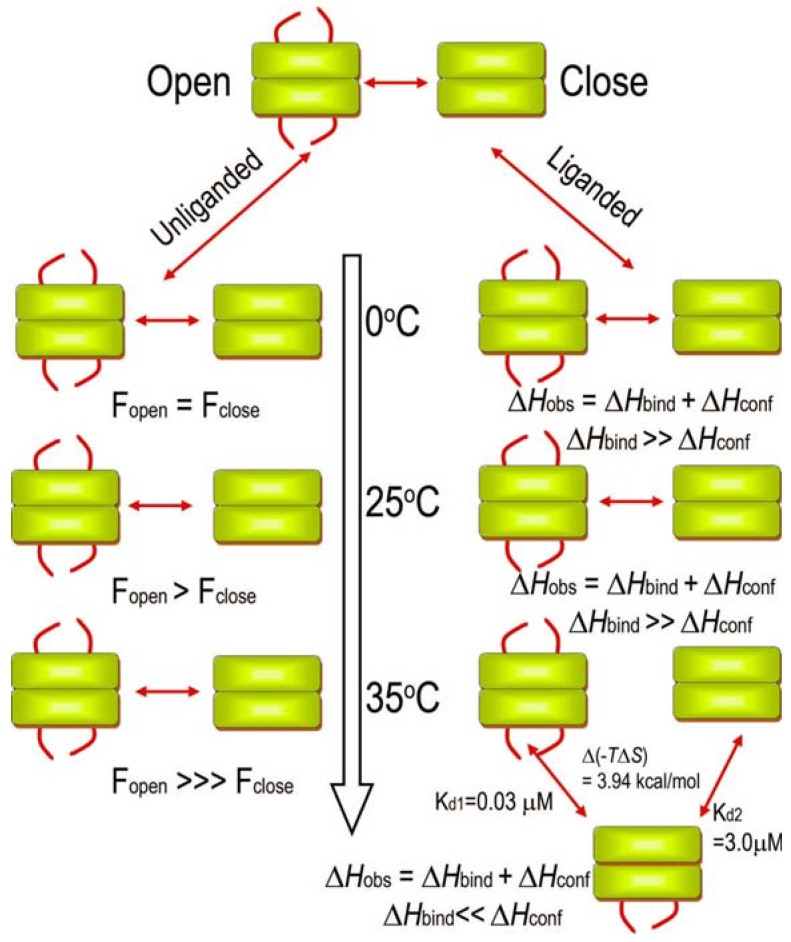
Schematic of the effect of temperature on the dynamics of the unliganded and liganded E1p [60]. In unliganded E1p (no MAP), the loops exist as a conformational equilibrium of open and closed states (Figure 4). This equilibrium gradually shifts in favor of the open conformation up to 25 °C; there is a step transition in favor of the open conformation at 25 °C. During ligand binding > 25 °C, the step “close↔open” transition gives rise to a configurational enthalpic term (Δ*H*_conf_) of a much higher magnitude, resulting from a ligand-induced disorder-to-order transition, which causes progressive reinforcement of observed enthalpy (Δ*H*_obs_). However, at 35 °C, where the rate of conformational fluctuations of the loop (*k*_ex_) and hence the coupled rate of covalent substrate addition (*k*_2_) increases, the catalytic rate is regulated by temperature induced anticooperative binding of ligand. At this temperature, binding of the second ligand incurs an entropic penalty, which results in 100-fold weaker affinity for the second molecule of ligand giving rise to the observed negative cooperativity.

For NMR purposes, the trifluoroacetonyl group was introduced [creating CF_3_C(=O)CH_2_SCys411-E1]. The enzyme activitiy is only slightly reduced and binding of ThDP and methyl acetylphosphonate (MAP) is not affected in these variants. ^19^F-NMR measurements were carried out for these constructs [60].^19^F-NMR has proven to be a powerful technique in the study of protein structure and dynamics, with no background signals. This ^19^F-NMR approach can be applied to the analysis of conformational states of protein molecules, which are too large or unstable for full NMR structure determination. The effect of exchanging conformations on the ^19^F resonance can directly determine the kinetic parameters of the conformational transition. The ^19^F spectra of K411C-TFA (K411C-derivatized) showed two distinct and unequally populated resonances at −8.993 and −9.106 ppm, assigned to the open and closed conformation of the loop, respectively. On addition of the substrate surrogate MAP, the resonance at −8.993 ppm disappeared and the one at −9.106 ppm became more intense (Figure 4). The effect of temperature on the ^19^F spectra on the unliganded K411C-TFA was carried out. The ratio of open to closed populations exhibited remarkable temperature dependence and resembled chemical exchange type effects [60]. The exchange rates (*k*_ex_ = *k*_AB_ + *k*_BA_) could be extracted by line-shape simulations. The exchange rate constant was found to be < 1.0 s^−1^. In order of magnitude, this value is similar to the observed *k*_cat_ of the K411C-TFA (*k*_cat_ = 0.38 s^−1^ according to E1-component-specific assay at 30 °C), suggesting a quantitative correlation of loop dynamics and catalysis in E1ec. These studies based on ^19^F-NMR measurements revealed that the overall dynamics of the E1p creates a regulatory switch to streamline the catalytic steps in the complex and the dynamics of the active centre loop may represent a rate limiting catalytic step (Figure 5). Our observations and conclusions might be of more general applicability for the other ODHc complex enzymes, which also most probably use dynamics to “fine tune” catalysis.

### 3.5. Nuclear Magnetic Resonance Evidence for the Role of the Flexible Regions of the E1 Component of the Pyruvate Dehydrogenase Complex from Gram-Negative Bacteria

In the X-ray structure of *E. coli* E1p with ThDP, three disordered regions lacking interpretable electron density were identified corresponding to the N-terminal 1–55 residues, and to stretch of residues 401–413 (inner active center loop) and 541–557 (outer active center loop) [5]. In a series of publications [59,60,61], the functional importance of the two active center loops in the organization of the active center during the reaction sequence and also probed the potential correlation of loop dynamics with enzyme catalysis was demonstrated (see previous paragraphs). In a sequel to these studies, the possibility that regions too mobile to be seen in the X-ray structure may give rise to resolvable resonances in the NMR spectrum, notwithstanding the size of E1p (2 × 886 residues for a *M*r of 200,000), was explored [62]. The total number of peaks in the ^15^N-^1^H HSQC TROSY spectrum was found to be 103 and correlates with the number of hydrogen resonances expected in the three flexible regions of E1 [(64 from the N-terminus, 19 for the inner (401–413) and 26 for the outer (541–557) active center loops for a total of 109] (Table 4). Sequence-specific NMR assignments were made for 6 residues in the N-terminal 1–55 region and for a glycine in each of the two mobile active center loops (G402 and G542) of the E1p component, by using strategies such as glycine auxotrophic strains and by comparison with the synthetic peptide which corresponds to N-terminal residues 1–32 of E1p.

With the assignment of residues serving as “reporters” for the three flexible regions of E1p, interaction studies were carried out with: (*a*) a stable pre-decarboxylation intermediate analogue known to affect the mobility of the active center loops, (*b*) an E2p-didomain construct comprising LD and PSBD, (*c*) a peptide corresponding to the amino acid sequence of the E2 PSBD and (*d*) lipoyl domain construct, to determine any changes in the mobility of these flexible regions upon interaction. The study in presence of a stable pre-decarboxylation intermediate that resembles LThDP in Scheme 2 (*k*_1_, *k*_2_), selectively broadens the NH resonances corresponding to G402 and G542, but not those resonances assigned to the N-terminal residues, proving that these two loops become organized, in accordance with both x-ray and dynamic measurements [59,60,61]. From the interaction studies of E1 with E2 components, it was found that none of the resonances from the N-terminal region of E1, which interact with either di-domain or PSBD were affected by the lipoyl domain. The evidence indicates that the N-terminal region of the E1p interacts mainly with the PSBD domain of E2, and this interaction precedes reductive acetylation. This is the first structural support for the hypothesis that the N-terminal region of E1 of this entire class of bacterial PDHc’s is responsible for binding the E2 component. Even though only 8 residues were identified out of 886 residues per E1p monomer, it is important to address the specificity of observations. The results clearly show that with such large proteins, the NMR and x-ray results reveal complementary views of structure and dynamics.

**Table 4:** Experimentally estimated and theoretical number of resonances on E1ec in the three flexible regions in the ^15^N-^1^H HSQC spectra [62].

	N-terminal region	Inner loop	Outer loop
Number of residues ^a^	55 (1–55)	13 (401–413)	17 (541–557)
Number of Gln	3	1	4
Number of Asn	2	2	1
Number of Pro	2	0	1
Number of Trp	1	0	0
Theoretical number of resonances	64	19	26
Number of resonances estimated (Experimental/Theoretical)	103/109 ^b^ 101/109 ^c^		
Number of resonances undetected on complexation	44 ^d^; 38 ^e^; 33 ^f^		

*^a^* Each backbone NH contributes one resonance. The side chains of glutamine and asparagine give rise to two resonances on two-dimensional HSQC spectra. For each proline present, the total number of resonances was decreased by one. The Trp gives rise to the indole NH resonance. *^b^* Spectrum of His_6_-tag E1p. *^c^* Spectrum of His_6_-tag E1p without ThDP. *^d^* Spectrum of His_6_-tag E1p in presence of E2 ^1−190^. *^e^* Spectrum of His_6_-tag E1p in presence of synthetic peptide corresponding to PSBD. *^f^* Spectrum of His_6_-tag E1ec in the presence of PLThDP.

## 4. Overall Architecture of the E2 Component Deduced from NMR Studies

The E2 lipoate acyltransferase components of the ODHc’s are highly segmented proteins, which form the core structures of the complexes. A combination of limited proteolysis and ^1^H-NMR experiments revealed the unusual quaternary structure of the E2 chains [63]. The 270 MHz ^1^H-NMR studies carried out on the entire *E. coli* PDHc in the early 80s revealed several sharp resonances between ‒0.6 and ‒3 ppm attributable to protein with line widths of only about 50 Hz (the expected line width for the enzyme complex is of the order of 8kHz). This clearly shows that there are substantial regions of the polypeptide chain (rich in Ala or Thr residues) with marked intramolecular mobility [63]. After treatment of the *E. coli* PDHc with trypsin, the most mobile regions were no longer observable without causing substantial dissociation of the complex, suggesting that the highly mobile regions are connected with the domain bearing the lipoyl-lysine. Similar results were obtained with the *E. coli* OGDHc complex [64]. From these observations it was inferred that, this novel conformational mobility, which is different from the substrate-induced conformational changes of enzymes, is a general property of the ODHc’s, and is most likely related to the active site coupling property possessed by these complexes.

Next, a stable proteolytic fragment (probable M_r_ 6500) of the E2 polypeptide chain from PDHc containing the lipoyl group was isolated. It appeared to have a robust secondary and tertiary structure indicated by its resistance to further proteolysis by several enzymes. ^1^H-NMR spectrum of this fragment corresponds to a well-defined folded structure, which remains unchanged up to at least 45 °C [65]. This lipoyl domain, unlike free lipoic acid, is a substrate for reductive acetylation by El. From electron microscopy, NMR and limited proteolytic studies on the intact PDHc complex, a rough idea of the overall structure of the PDHc complex was obtained. The lipoylated regions protrude from an inner region of the E2p core between the Elp and E3 components and can be released from the PDHc by limited trypsinization at Lys-316 without causing disassembly of the particle. The same result was obtained with V8 proteinase treatment. Harsher treatment of the complex with the same proteinase brought about an additional cleavage of E2p at Glu-372 [66], leading to the dissociation of the E3 component but not of Elp. This suggested that the region of E2p between Lys-316 and Glu-372, may represent an additional folded domain (probably PSBD) which is of particular importance in E3 binding [67].

**Figure 6:**
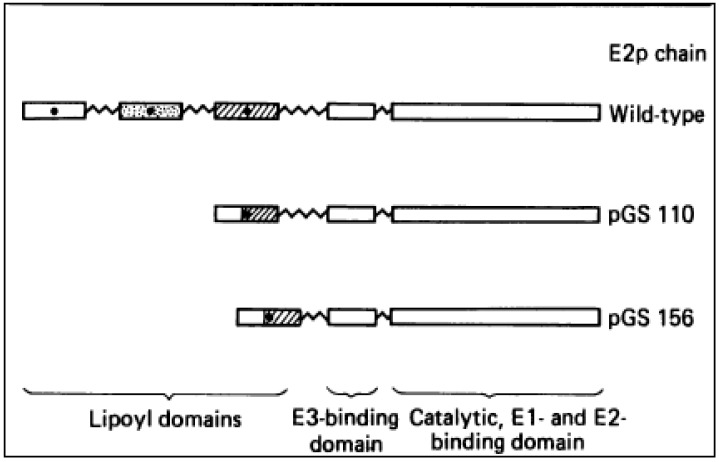
Schematic representation of the structure of the wild type, pGS110- and pGS156encoded E2p chains.

Genetically reconstructed *E. coli* PDHc missing two of the three homologous lipoyl domains revealed the absence of many of the unusually sharp resonances previously attributed to flexible segments in the wild-type E2p polypeptide chains [68]. Examination of the spectra revealed another sharp but smaller resonance at 1.52 ppm in the single lipoyl PDHc (pGS156) complex; in addition to the 1.32 ppm peak corresponding to long flexible (Ala+Pro) rich linkers; which was not apparent in the other wild type and 2-lipoyl domain complex. This additional resonance clearly arose from the E2p chains; as the released E3 or E1p fragment did not exhibit this peak. Hence; an additional smaller; (Ala+Pro) rich linker sequence separating the E3 binding domain from the inner core domain in the C terminal half of the E2p chain was identified.

This also provides an explanation for fluorescence data which suggested that E3 within the PDHc is quite mobile [69]. These experiments provided an explanation for active site coupling which likely has its origins in the highly segmented structure of the E2p polypeptide chain. The number of lipoyl domains per E2 chain ranges from one to three. The lipoyl domain (or domains) (LD) is followed by a small (~35-residue) PSBD, and a much larger (28 kDa) catalytic domain with acyltransferase activity and aggregates to form the octahedral (24-mer) or icosahedral (60-mer) inner core of the ODHc. These domains are separated by 25–30 amino-acid residue long segments of polypeptide chain, which act as extended, flexible linkers that facilitate domain movement as part of the catalytic mechanism. This architecture of E2 can be generalized for all the ODHc complexes.

### 4.1. Solution Structures of the N-Terminal Lipoyl Domains of E2

**Figure 7:**
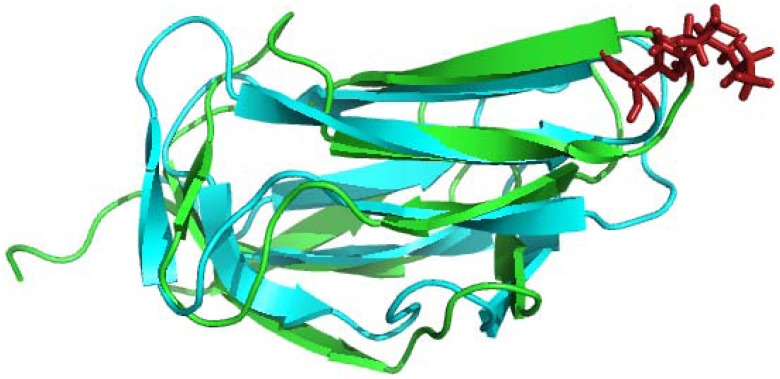
Overlapped lipoyl domain structures of innermost lipoyl domain of the PDHc complex (Green; PDB Id:1QJO) [81] and lipoyl domain OGDHc complex from *E. coli* (Cyan; PDB Id: 1PMR) [14]. Lipoyl lysine residue is highlighted in stick form. The image was created using the program PyMOL.

After development of multidimensional NMR approaches, structure determination of lipoyl domains from different species was carried out by several groups [12,13,14,15,70,71]. Lipoyl domains are small (8 kDa) substrate carrying domains of the ODHc’s. The overall structures of lipoyl domains of known structure from many species possess many common features (Figure 7): (1) eight beta strands arranged in two antiparallel sheets in the form of a flattened beta barrel with a 2-fold quasi symmetry axis. The first sheet consists of β strands 1, 3, 6 and 8, whereas the second is formed by β strands 2, 4, 5 and 7 and each sheet contains three major strands and one minor strand (β 3 and β 7, respectively) (2). The N and C-terminal residues are close together in space in the β-sheet on one side of the domain (3). The lipoyl-lysine residue is prominently displayed at the tip of a type1 β-turn in the second β-sheet opposite to that which contains the N- and C-termini. This lipoyl-lysine side-chain forms a so-called ‘‘swinging arm’’, which helps to shuttle the acyl group between active sites of E1 and E2 in the assembled complex. The interior core of the β-barrel is packed with hydrophobic residues, and most of these hydrophobic residues are conserved in all lipoyl domains. The structures of the lipoyl domains throw light on the two important recognition processes, lipoylation of specific lysine residue by the lipoylating ligase enzyme [72] and recognition by the cognate E1 for the specificity of reductive acylation of their pendant lipoyl groups, an important aspect of the mechanisms underlying active site coupling and substrate channeling [73].

In the case of mammalian PDHc and BCKDHc, the lipoyl domains also play an important role in regulation [74,75,76,77]. Their activities are regulated post-translationally by phosphorylation/dephosphorylation of E1 chain by kinases and phosphatases, respectively, under various dietary conditions and hormonal stimuli [76,78,79,80]. Although the role of the PDKs is to down-regulate the activity of the E1 component, the kinases appear to have much greater binding affinity for the E2/E3BP component, offering an additional degree of regulation on this key complex. In order to obtain thorough interaction maps between human E2 and the PDKs, sequence-specific NMR resonance assignments of the N-terminal L1 domain, L2S didomain (inner lipoyl domain L2 + PSBD abbreviated as S here) and the L1L2S tridomain (L1 + L2 + S) constructs of the human pyruvate dehydrogenase E2 component, L3S didomain (L3 + S) of the E3BP are being carried out in our groups. Even though there is high sequence similarity between the lipoyl domains, the residues from the L1 and L2 domains do not overlap and their resonances could be assigned using ^15^N,^13^C doubly-labeled L1L2S proteins.

**Figure 8:**
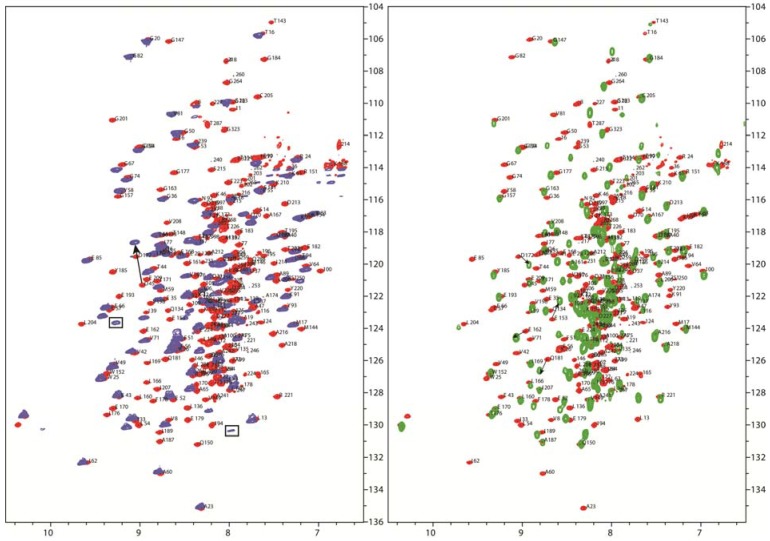
^15^N-^1^HHSQC-TROSY spectra of L1L2S (red peaks) overlaid with L1 (blue peaks) (**Left**) and L2S (green peaks) (**Right**). Two residues from the L1 domain which disappeared in the L1L2S construct are indicated with a box. Residues which show large chemical shift change are indicated with arrows. Assigned peaks are indicated with one letter amino acid code and residue number.

When we overlaid the ^15^N-^1^H HSQC-TROSY spectra of L1 and L2S with the larger tri-domain (L1L2S) construct, most of the residues occupy similar positions, even though there is slight chemical shift difference owing to the different sample conditions, indicating that their overall conformation is unaltered. Two residues L10, Q18 (boxed in Figure 8) which could be assigned in the L1 domain disappeared in the larger L1L2S tri-domain construct and a few negatively charged residues, such as D45, E153, E162, D172, Q181 display larger chemical shift changes (indicated by black arrows in Figure 8). All of these residues are located in the flexible loop regions of the lipoyl domains. These residues might participate in electrostatic interactions with the PSBD, which interaction may be competed with by the presence of the other lipoyl domain in the larger L1L2S construct.

### 4.2. Positioning of the Lipoyl Lysine Residue for Correct Post-Translational Modification

In all ODHc’s a lipoyl group is attached through covalent amide linkage to a specific lysine *N*6-amino group side chain located at the tip of a *β*-turn in the lipoyl domain. Highly conserved Asp and Ala residues flank this Lys residue in all lipoyl domains. To investigate the role of the highly conserved DKA motif in the type1 *β*-turn region, site directed mutagenesis studies were carried out in the *Bacillus stearothermophilus* PDHc complex. Singly (D41A, D41E, D41K, A43M, A43E, A43K) and doubly substituted variants (D41MA43M, D41KK42A) were tested for lipoylation specificity and acylation efficiency [72]. It was found that only the correct target lysine was lipoylated and double lipoylation was not found in D41K, A43K constructs. Likewise in case of D41KK42A, where the original Lys was replaced, there was no lipoylation despite the proximity of the new Lys. All substitutions at position D41decreased the rate of reductive acylation. At position A43, substitution with larger residue (A43M) did not affect the rate, but substitution with charged residues (A43E, A43K) led to a decreased rate of reductive acylation. From these studies it is inferred that correct positioning of the target lysine at the tip of a *β*-turn is of crucial importance, but not as much of the conserved Asp and Ala residues adjacent to the lipoyl lysine residue [72]. However, recognition by E1 for acylation appears to be dependent on the side chains of the residues surrounding the lipoyl lysine. It was shown by ^1^H-NMR that acetylation occurs at the S8 position of the lipoic acid and not at the S6 position, although isomerization of the acetyl group can occur in aqueous solution [82].

### 4.3. Solution NMR Structure of Peripheral Subunit Binding Domain

**Figure 9:**
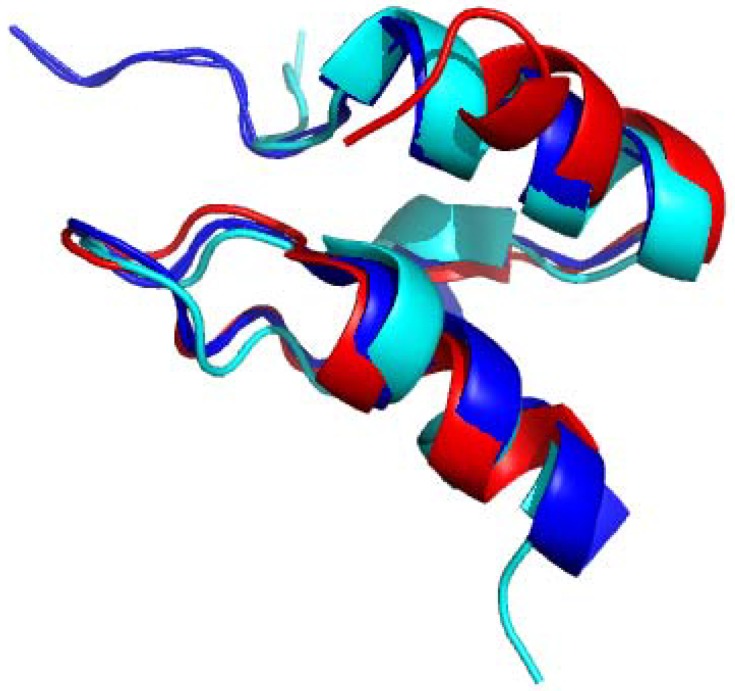
Overlaid structures of PSBD from OGDHc complex of *E. coli* (Red; PDB Id:1BBL) (*16*) and PDHc complex of *Bacillus stearothermophilus* (Cyan; PDB Id:1W3D) [86] and peripheral-subunit from mesophilic, thermophilic and hyperthermophilic bacteria fold(Blue; PDB Id:1W4H) [84]. The image was created using the program PyMOL.

The PSBDs are integral parts of E2 components, which bind to the peripheral E1 and E3 components within the ODHc’s. They are the smallest globular protein domain known without disulfide bridges, metal ions, or prosthetic groups. Their surface is characterized by the presence of large number of positively charged residues and they exhibit varying degrees of ionic strength dependent characteristics [83,84]. These charged residues are required for binding to catalytic peripheral subunits via electrostatic interactions. Solution structures of PSBD domains of E2 components from different ODHc’s have been solved by using synthetic and recombinant peptides [16,17,84,85]. The structures of the PSBDs are very similar, consisting of approximately 35 residues and form two parallel *alpha* helices, separated by a short strand, a helix-like turn and an irregular loop, with a hydrophobic core (Figure 9). A cluster of basic residues in and around Helix I of PSBD causes significant electrostatic strain and is susceptible to ionic strength effects [83] and some of these basic residues constitute part of the proposed binding site that appears conserved in the PSBD family. From these studies few general criteria on the E2-E3 interface can be assessed. It seems that electrostatic interactions (salt bridges and/or hydrogen bonds) are likely to be particularly prominent features of the E2-E3 interface and these interacting residues should have highly solvent accessible side chains. Since there is only one E3 chain for all ODHc complexes within a single organism, it is likely that contact residues are highly conserved in E2 components.

### 4.4. Role of Extended Polypeptide Linkers as Structural and Functional Determinants

Cryoelectron microscopy studies showed that the annular gap of ~75–90° between the E2 core and the shell of peripheral enzymes is maintained by the flexible but extended conformation adopted by 60 linker polypeptides that radiate outwards from the inner E2 core, at each 3-fold vertex of the E1 or E3 occupancy [87,88]. From NMR analysis, using synthetic peptides corresponding to the PDHc inner linker region, it was suggested that Pro residues of the inner *B. stearothermophilus* PDHc linker are primarily in the all-trans conformation, probably enhancing elongation of the peptide and providing some degree of rigidity, while their conformational flexibility facilitates productive interactions [89]. Similar results were obtained with synthetic peptides corresponding to the three Ala-Pro rich inter domain sequences (20–30 residues long) that link the lipoyl domains to each other and to the PSBD in the *E.* *coli* E2p chain. It was also found that the innermost of the three linkers between the lipoyl domains has the highest proportion of Ala-Pro sequences and is responsible for keeping the lipoyl domains from collapsing onto the PSBD and the inner core of the PDHc complex [90,91].

### 4.5. NMR Studies of Interaction of PDHc Component Enzymes Using Truncated Domain Constructs

**Scheme 5:**
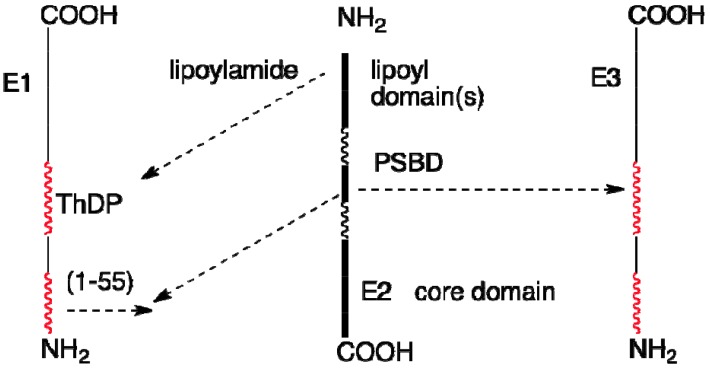
Interactions among components in the *E. coli* pyruvate dehydrogenase complex; more on E2-E3 interactions in ref. [95].

NMR interaction studies between the N terminal fragments of E2o and E2p chains with their peripheral enzymes has been carried out by two groups [92,93,94,95,96]. In Scheme 5 are indicated the known interactions among components of the *E. coli* PDHc.

In the case of *B. stearothermophilus* PDHc, the interaction on E2p with E3 was limited almost exclusively to residues Asp41 and Ala43, part of the DKA motif anchor of the lipoyl lysine residue and no domain specificity was observed. NMR examination of the interaction of the *E. coli* PDHc N-terminal E2p didomain (comprising a hybrid lipoyl domain and PSBD) and E3, revealed negligible changes in chemical shifts, detecting little or no interaction between E3 and the lipoyl domain [95].

**Figure 10:**
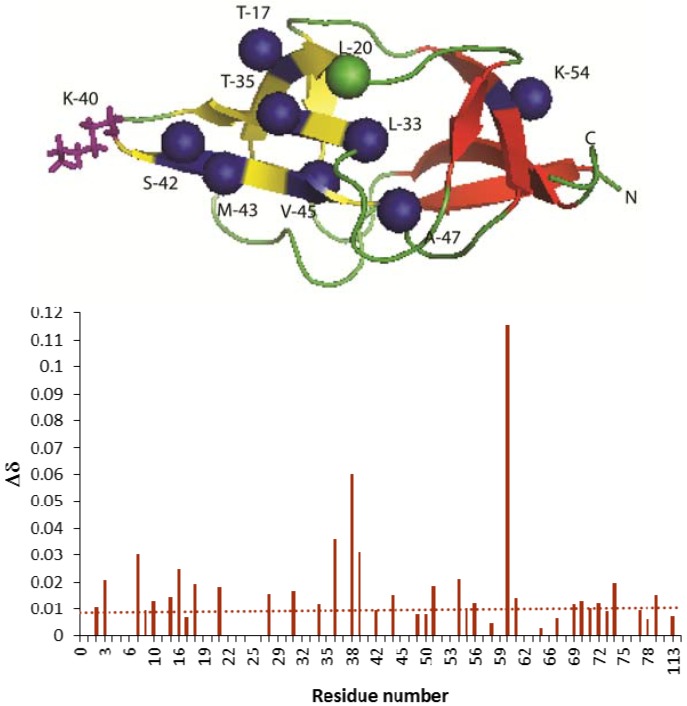
(**Top**) Structure of inner lipoyl domain from *E. coli* PDHc (PDB Id:1QJO). E2p- didomain residues, which disappeared on complexation with E1p were represented as spheres. Blue spheres represent the same amino acid in both the hybrid lipoyl domain and inner lipoyl domain. Green spheres correspond to different amino acids in the hybrid lipoyl domain (L-20 instead of M-21). Lipoyl lysine residue side chain in the type1 β turn is represented as a stick model. (**Bottom**) Chemical shift deviation of E2p-didomain after addition of 1 equivalent of E1p in the presence ThDP and pyruvate.

In contrast NMR investigations of E2-E1 interactions revealed that the prominent surface loop that links the first and second *β*-strands of the lipoyl domain, which lies close in space to the lipoyl-lysine *β*-turn, is an important determinant of its interaction with E1p in various ODHc complexes. The residue immediately adjacent to the lipoyllysine motif (DKA/V) was also thought to be involved in specifying the interaction with E1 [92,93,94,96] in *B. stearothermophilus* PDHc. In the case of hybrid lipoyl domain *E. coli* PDHc, the major residues, which disappeared upon interaction with E1p were mainly located in the β strands 4 and 5, which connects the lipoyl-lysine β-turn (Figure 10). However, distinct chemical shift changes (>0.01 ppm) were observed for residues centered around the lipoyl lysine β-turn region and from the surface loop region on the domain between β-strands 1 and 2, which lies close in space to the lipoyl-lysine β–turn. From these studies, it was clear that E1p components recognize residues distributed chiefly over the half of the domain that contains the lipoyllysine residue to ensure a productive interaction. However, a mosaic of other contacts over the entire lipoyl domain is also observed. Although the surface loop and the lipoyllysine turn residues are likely to play significant roles, either by direct interaction or by maintaining the domain in the correct conformation, they are not the sole determinants of this recognition process.

### 4.6. Dynamics Studies of the Truncated E2 Component Constructs

Proteins are known to be dynamic, and they sample numerous conformational states. The role of active site flexibility in enzyme catalysis has been emphasized [97,98]. Each catalytic event requires a minimum of three or more steps and is exquisitely dependent on spatial and temporal changes in bio macromolecules occurring on a wide range of time scales. In addition to the structures, NMR spectroscopy can yield information on the dynamic properties of molecules over a range of different time scales with atomic resolution [99,100,101,102]. This usually involves measuring relaxation times such as T_1_ and T_2_ to determine order parameters, correlation times, and chemical exchange rates. Perham and co-workers (21) and Chuang *et al.* [15,85] have measured the ^15^N-NMR relaxation parameters of lipoyl domain from *E. coli* PDHc and lipoyl domain(LD), PSBD and didomain (LD+PSBD) from human BCKDHc.

**Figure 11:**
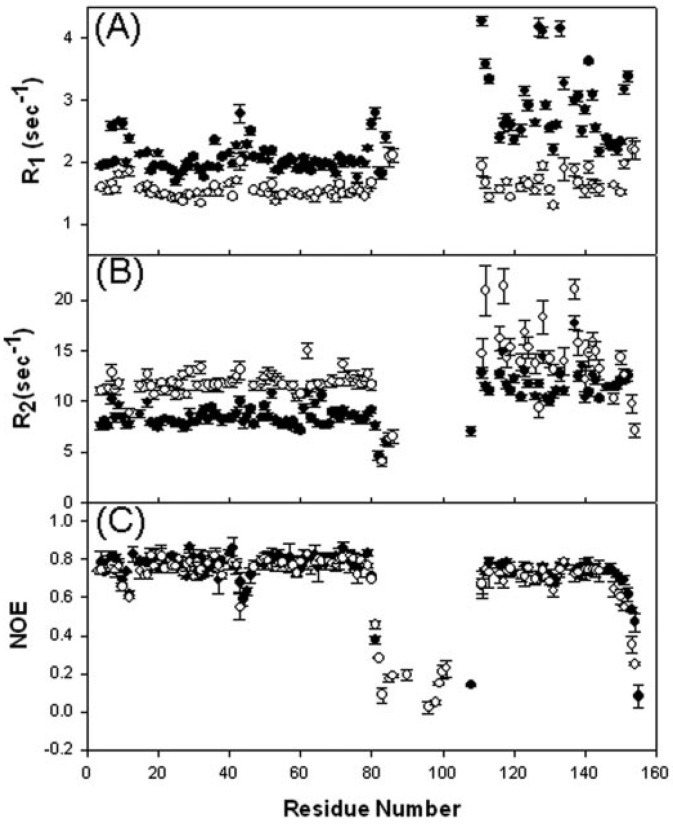
Sequence-dependent variations of the amide ^15^N-R1 (**A**), ^15^N-R2 (**B**), and ^1^H-^15^N NOE (C) of LD (*filled circles*), PSBD (*filled circles*), and di-domain (*open circles*) of human BCKDHc [85].

From these studies it was shown that the residues in and surrounding the lipoyllysine β-turn are highly mobile and become less flexible after lipoylation of the lysine residue. The relaxation data profile of the free lipoyl domain and the linked LD in the di-domain are nearly identical indicating the internal dynamics remains unchanged. However, the *R*2 values of LD in the di-domain are higher than those of isolated LD, and the situation is reversed for the *R*1, due to the increase in the rotational correlation time in the di-domain (Figure 11). On the other hand, PSBD exhibits higher dynamic nature than LBD both as a free domain and as a moiety of the di-domain and they rotate more slowly than the larger LBD. The data also suggested that the linker imposes significant rotational constraints on the two constituent domains such that LD and PSBD in the di-domain construct are not rotating freely and independently of each other in the solution. The overall correlation time of LD and PSBD changed from 5.54 ns and 5.73 ns in isolated forms to 8.37 ns and 8.85 ns in the di-domain construct [85].

## 5. Conclusions

OGDHc and PDHc complexes are key members of the primary energy-producing pathways of glycolysis and the tricarboxylic acid cycle, likewise the branched chain 2-oxoacid dehydrogenase plays important role in the catabolic pathways of valine, isoleucine, and leucine metabolism. So far it has proved impossible to crystallize any intact E2 component. However, NMR spectroscopy could provide high-resolution conformational structure determination of the domain and linker structures of E2 components. Ongoing studies indicate that with the highly organized lipoyl domains, sequence-specific assignments should be obtainable on multiple domains of the E2 components (so far, we have found this to be the case for the human pyruvate dehydrogenase complex), suggesting a promising future for NMR interaction studies among components. On the other hand, it is also the case, that with so many domains in play, it is important to develop methodologies for studying the structure and interactions of intact E2 components, currently outside the capabilities of solution NMR methods. Recognizing these limitations of NMR, we have started to use mass spectroscopic methods to address similar issues and inter-component interactions in these complexes. NMR methods have also been applied to obtain information on the ionization and tautomerization states of ThDP bound to three enzymes as well as in model compounds by monitoring the key carbon and nitrogen atoms specifically introduced into the coenzyme.

## Supplementary Materials

Supplementary File 1
