# Supplementary material for: Nuclear Magnetic Resonance Approaches in the Study of 2-Oxo Acid Dehydrogenase Multienzyme Complexes—A Literature Review
Source: Molecules. 2013 Sep 26;18(10):11873–903. doi: 10.3390/molecules181011873 (PMC6270654; doi:10.3390/molecules181011873)

Domain structure of E2 component from *E. coli* pyruvate dehydrogenase complex

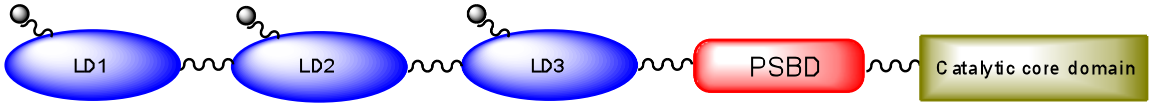

LD: Lipoyl Domain   PSBD: Peripheral Subunit Binding Domain

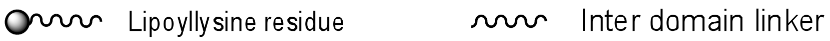

Supplement: Supplementary File 1 [file molecules-18-11873-s001.pdf]
